# Oncogenic Role of an Epigenetic Reader of m^6^A RNA Modification: YTHDF1 in Merkel Cell Carcinoma

**DOI:** 10.3390/cancers12010202

**Published:** 2020-01-14

**Authors:** Elias Orouji, Wiebke K. Peitsch, Azadeh Orouji, Roland Houben, Jochen Utikal

**Affiliations:** 1 Skin Cancer Unit, German Cancer Research Center (DKFZ), Im Neuenheimer Feld 280, 69120 Heidelberg, Germany; 2 Department of Dermatology, Venereology and Allergology, University Medical Center Mannheim, Ruprecht-Karl University of Heidelberg, Theodor-Kutzer-Ufer 1-3, 68167 Mannheim, Germany; wiebke.ludwig-peitsch@vivantes.de (W.K.P.); azadeh.orouji@umm.de (A.O.); 3 Department of Genomic Medicine, University of Texas MD Anderson Cancer Center, Houston, TX 77054, USA; 4 Department of Dermatology and Phlebology, Vivantes Klinikum im Friedrichshain, Landsberger Allee 49, 10249 Berlin, Germany; 5 Department of Dermatology, Venereology and Allergology, University Hospital Würzburg, Josef-Schneider-Straße 2, 97080 Würzburg, Germany; houben_r@ukw.de

**Keywords:** Merkel cell carcinoma, Merkel cell polyomavirus, copy number variations, m^6^A, RNA modification, epitranscriptome, YTHDF1, epigenetic reader

## Abstract

Merkel cell carcinoma is a deadly skin cancer, which in the majority of cases is caused by the Merkel cell polyomavirus (MCPyV). The viral small T antigen is regarded as the dominant oncoprotein expressed in the tumor cells. We used genomic screening of copy number aberrations along with transcriptomic analysis to investigate regions with amplification that harbor differentially expressed genes. We identified YTHDF1, a protein that is a reader of N^6^-methyladenosine (m^6^A) RNA modifications, to have high copy gains and to be highly expressed in Merkel cell carcinoma. Importantly, we identified the presence of m^6^A on small T antigen mRNA suggesting a relation between YTHDF1 amplification and MCPyV gene expression. Interestingly, knockdown of YTHDF1 in Merkel cell carcinoma (MCC) cell lines negatively affected the translation initiation factor eIF3 and reduced proliferation and clonogenic capacity in vitro. Furthermore, analysis of survival data revealed worse overall survival in YTHDF1^high^ MCC patients compared to YTHDF1^low^ patients. Our findings indicate a novel oncogenic role of YTHDF1 through m^6^A machinery in the tumorigenesis of MCC.

## 1. Introduction

Merkel cell carcinoma (MCC) is a highly aggressive and rare skin cancer which is twice as lethal as malignant melanoma [1]. In about 80% of MCC cases the Merkel cell polyomavirus (MCPyV or MCV) is detected [2] and the finding that it is clonally integrated in the genome of the tumor cells [2] is one of the main arguments that MCPyV is the crucial causal factor for virus-positive MCCs. MCPyV is a member of the Polyomaviridae family of viruses, 13 types of which can infect humans [2]. MCPyV is related to the animal tumor virus simian virus 40 (SV40), and like in the case of SV40, oncoproteins named T antigens are generated by differential splicing of a common mRNA producing sequences coding for large T (LT), small T (sT), and 57 kT antigens [3]. It has long been known that SV40 late mRNAs—encoding the viral capsid proteins—contain N^6^-methyladenosine (m^6^A) residues in their sequence [4,5]. However, presence of m^6^A modification has so far not been reported for any of the MCPyV mRNAs. Amongst the known RNA modifications in eukaryotes, m^6^A is one of the most abundant internal (non-cap) modification of mRNAs and contributes to RNA structure, localization, and function [6,7]. m^6^A is present in the RNA transcripts of viruses with nuclear replication, including influenza A virus, SV40, Rous sarcoma virus, avian sarcoma virus, and adenovirus [8,9,10,11,12]. It is involved in the regulation of many different biological processes such as gene expression, mRNA splicing, processing and stability, apoptosis, microRNA biogenesis, stem cell differentiation, and cancer [13,14].

The m^6^A machinery is composed of a series of proteins including methyltransferases (m^6^A writers) that work along with cofactors, enzymes whose role is to demethylate m^6^A (m^6^A erasers), and those that recognize m^6^A in the RNA transcript (m^6^A readers). m^6^A modifications in the 3′ untranslated region (3′ UTR) can be recognized by either YTHDF1 or YTHDF2. YTHDF1 specifically binds to m^6^A-containing mRNAs and promotes cap-dependent translation [15]. Furthermore, YTHDF1 was shown to enhance loading of ribosomes to m^6^A-containing mRNAs as well as to interact with translation initiation factors eIF3A or eIF3B [16]. Interestingly, YTHDF2 which also binds to m^6^A-containing mRNAs is functionally divergent. Its role is to facilitate mRNA decay through relocation of RNA to the degradation sites of the cell [17]. YTHDF2 has recently been shown to have several thousands of RNA targets, including mRNA as well as non-coding RNAs [17]. The function of a further family member (i.e., YTHDF3) is still not fully studied. This study aimed to elucidate the role of m^6^A readers, particularly YTHDF proteins in the tumorigenesis of Merkel cell carcinoma.

## 2. Materials and Methods

### 2.1. Patient Samples and MCC Cell Lines

This study used a panel of cell lines derived from patients with histologically confirmed MCC diagnosis. MCPyV^positive^ cell lines included PeTa, WaGa, MKL1, MKL2 [18,19,20], and MCPyV^negative^ cell line includes UISO, MCC13, MCC26 [21,22,23,24]. MCC cell lines were cultured in RPMI-1640 supplemented with 10% fetal bovine serum, 100 U/mL penicillin, and 0.1 mg/mL streptomycin. Tumor tissue specimens, from a cohort of 31 MCC and 26 melanoma patients, in formalin-fixed paraffin-embedded (FFPE) blocks along with tissue microarrays of these samples were investigated in this study. Samples were gathered from patients referred to the Mannheim Department of Dermatology, Venereology and Allergology, Heidelberg University during 2001–2015 (ethical committee vote: 2014-835R-MA). All the MCC patients were MCPyV^positive^.

### 2.2. Immunohistochemistry (IHC) and Western Blot

Formalin-fixed paraffin-embedded blocks of MCC tumors were cut into 5 µm sections. Sections were pretreated by rehydration followed by antigen epitope retrieval using citrate buffer. IHC was performed using standard protocols provided by the manufacturer. Anti-YTHDF1 antibody (Abcam, ab99080, Cambridge, MA, USA) and anti-YTHDF2 (Abcam, ab88809) were used for immunostaining as well as Western blotting. IHC sections were scored using our previously described method [25]. Analysis of IHC sections was performed by two independent investigators.

For Western blot, cells were lysed in Radioimmunoprecipitation assay (RIPA) lysis buffer (Thermofisher Scientific, Waltham, MA, USA) and centrifuged at 15,000× *g* for 20 min at 4 °C. Protein was assayed using a Pierce BCA Protein Assay Kit according to the manufacturer’s protocol. Then, 30–100 μg of protein was run on an SDS polyacrylamide gel. Then membranes were blocked for 1 h at room temperature with Odyssey blocking buffer (LI-COR, Lincoln, NE, USA). Then, the membranes were incubated with the primary antibodies (anti-YTHDF1, Abcam, ab99080; anti-YTHDF2, Abcam, ab88809; anti-β-actin, Cell Signaling (Danvers, MA, USA), 5125S) overnight at 4 °C followed by 1 h incubation at room temperature with IRDye 800 secondary antibodies (LI-COR). The membranes were washed three times in PBS containing 0.01% Tween-20 for 5 min between each step. Blots were scanned, and proteins were detected using Odyssey Imaging System (LI-COR).

### 2.3. Gene Expression Analysis and Copy Number Analysis

Total RNA was isolated from cell lines using RNeasy Mini Kit (Qiagen, Germantown, MD, USA) per the manufacturer’s protocol. RNA samples were measured using Agilent 2100 Bioanalyzer, Santa Clara, CA, USA. Gene expression profiling was carried out using Illumina whole genome BeadChip Sentrix array, HumanHT-12 v4 platform (San Diego, CA, USA). Data was normalized and analyzed using Chipster 2.9.X. False Discovery Rate (FDR) < 0.05 was used as statistical significance throughout the analysis.

Copy number analysis was performed in MCC cell lines using Illumina Infinium CytoSNP-12 BeadChip which is a panel of ~300 k genome-wide tag single nucleotide polymorphism (SNPs) targeting regions of cytogenetic aberrations. Data was analyzed using Nexus Copy Number™ v 7.5, a software to detect and visualize genomic alterations.

### 2.4. m^6^A Distribution Prediction

Prediction score of m^6^A distribution across MCC cell lines were determined using the sequence-based RNA adenosine methylation site predictor (SRAMP) algorithm developed by Zhou et al. This tool is available online [26].

### 2.5. m^6^A Methylated RNA Immunoprecipitation (meRIP)

RNA was extracted from the cells using the RNeasy Mini Kit (Qiagen) according to the manufacturer’s instructions. RNA was then fragmented using zinc fragmentation buffer (10 mM ZnCl2, 10 mM Tris-HCl, pH 7.0). Reaction mix was incubated at 95 °C for 5 min, followed by inactivation with 50 mM EDTA and then was placed on ice. Fragmentation was followed by ethanol precipitation. Anti-m^6^A antibody (Abcam, ab99080) and rabbit IgG were crosslinked to the Dynabeads (ThermoFisher Scientific). MeRIP mix was prepared with 50 μg of the fragmented RNA in 500 μL of binding buffer plus 500 U of RNase inhibitor and incubated 1 h at room temperature. Non-crosslinked fragmented RNA was used as input. MeRIPs were washed with binding buffer at room temperature. Then, RNA was eluted from the beads by elution buffer at 42 °C. Next, cDNA synthesis was performed according to the SuperScript III First-Strand Synthesis System (Life Technologies, Camarillo, CA, USA) protocol. cDNA was then used for qPCR using SYBR Green. Two primer pairs were designed for each m^6^A site as well as a negative region. qPCR data for each m^6^A site were calculated using the ΔΔCt approach taking the negative site for normalization. Sequence of qPCR primers used to validate predicted m^6^A sites upon methylated RNA immunoprecipitation: Site1_fwd: GGAATTGAACACCCTTTGGAGC; Site1_rev: TAAGCATGCACCCAGGACC; Site2_fwd: TCCCATCTAGGTTGACGAGG; Site2_rev: GATCTTGAGTTGGTCCCGTGT; Site3_fwd: TCTTCCTCTGGGTATGGGTCC; Site3_rev: GGTCTCCTCTCTGCTACTGGA; Site4_fwd: TGAATATGAGCTAGACGACCACT; Site4_rev: CCTGGTCATTTCCAGCATCTCT; Site5_fwd: GCCTGATACAACCTTTAAGCCT; Site5_rev: GGGCCCTCTTCCTCAATAAGAA; Site6_fwd: GGGCCCACTCCATTCTCATC; Site6_rev: AGTATGGTGTCCTGATCCTTCT; Site7_fwd: TGCAAATCCAGAGGTTCTCCC; Site7_rev: CATTGCAGATGTGGGAGGCAA; Site8_fwd: AAACTGTTCAGCTGTGAACCC; Site8_rev: TACTGAACTAAGTGCCACCAC; Neg_Ctrl_fwd: GAGGCTCTCTGCAAGCTTTT; Neg_Ctrl_rev: TGGAATTTGCTCCAAAGGGTG.

### 2.6. shRNA-Mediated Knockdown

Lentiviral backbone for non-targeting shRNA (pLKO.1) and shRNAs against YTHDF1 (sh01: TRCN0000062772, sh02: TRCN0000062771) and YTHDF2 (sh01: TRCN0000168709, sh02: TRCN0000168751) were purchased from RNAi Consortium shRNA library, Broad Institute, Cambridge, MA, USA. Lentiviral particles were generated as described previously. WaGa and PeTa cells were transduced and selected using blasticidin (Invitrogen, Waltham, CA, USA) at different concentrations, based on the cell type. Knockdown efficiency was determined using YTHDF1 and YTHDF2 qPCR as well as Western blot. YTHDF1_fwd: TGTTCATGAAGCATGTCGGC; YTHDF1_rev: GCGGGTAATAGCTGGACAGG and YTHDF2_fwd: GCTCTTGGGCTAGAGCGTC; YTHDF2_rev: CGACATGGCTCTCAGATCCTC were used to assess expression levels of these genes.

### 2.7. Proliferation and Colony Formation Assay

Cells were seeded in triplicate, in 96-well plates and 180 µL of RPMI-1640 media was added to the wells. Plates were maintained at standard culture conditions of 5% CO_2_ at 37 °C. Alamar blue (20 µL) was added to the cells resulting in a final 10% solution. Fluorescence was measured at Ex:560 nm and Em:590 nm using Tecan Infinite 200 spectrophotometer (Tecan Life Sciences, Männedorf, Switzerland).

To perform the clonogenicity assay, 2000 cells were plated in each well of a 24-well culture plate and incubated 3–5 days at 37 °C. Media was removed and colonies were fixed and stained with 0.6% *w/v* methylene blue in methanol. The experiment was repeated 3 times.

### 2.8. Quantitative RT-PCR

Total RNA was isolated using the RNeasy mini kit (Qiagen). cDNA synthesis was performed using the SuperScript III First-Strand Synthesis System (Life Technologies). PCR quantification was performed using the SYBR Green method. Following primer sequences were used for eIF3A_fwd: AGAGTAGAGCGCCTGTACCA; eIF3A_rev: TGGTTATGGTGGCGCTGAAT; eIF3B_fwd: AGTACCGGAAAATGGCCCAG; eIF3B_rev: TGCTCCAGGTCACTCCTGAT; eIF4E_fwd: GACTGTCGAACCGGAAACCA; eIF4E_rev: AAACTTGGAGATCAGCCGCA; eIF4G_fwd: GGCAGGCGTATCCTGTGTG; eIF4G_rev: GGTGAGGGTATCAACCTGGC; eIF4H_fwd: GGACTTCGACACCTACGACG; eIF4H_rev: GTCGCCCTGAACCGTATTGA; GAPDH_fwd: CCTGCACCACCAACTGCTTA; GAPDH_rev: GGCCATCCACAGTCTTCTGAG.

The efficiency of qPCR primers for each primer pair was determined by serial dilutions of cDNA. Samples were normalized to GAPDH using the ΔΔCt analysis.

## 3. Results

### 3.1. YTHDF1 Is Highly Amplified and Expressed in Merkel Cell Carcinoma Cell Lines

**Figure 1:**
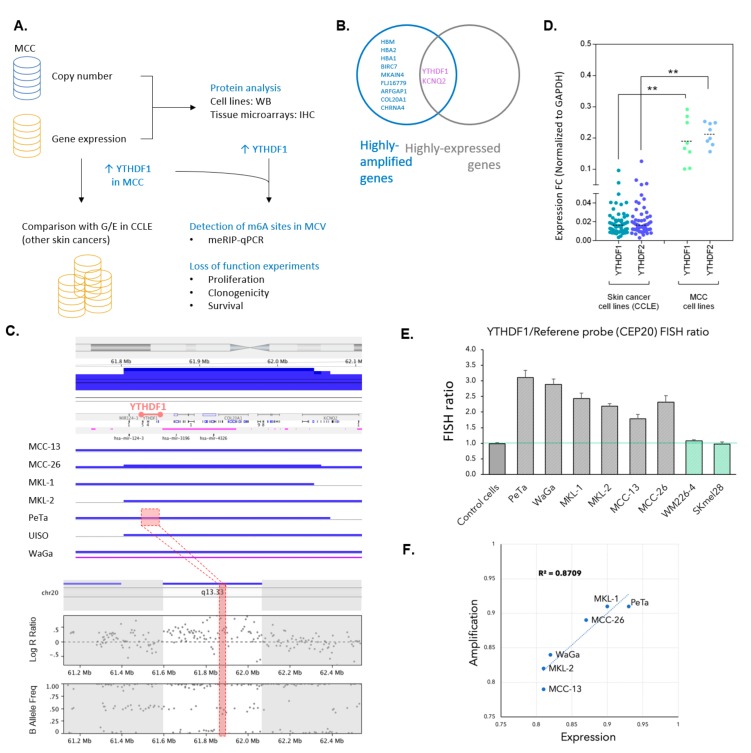

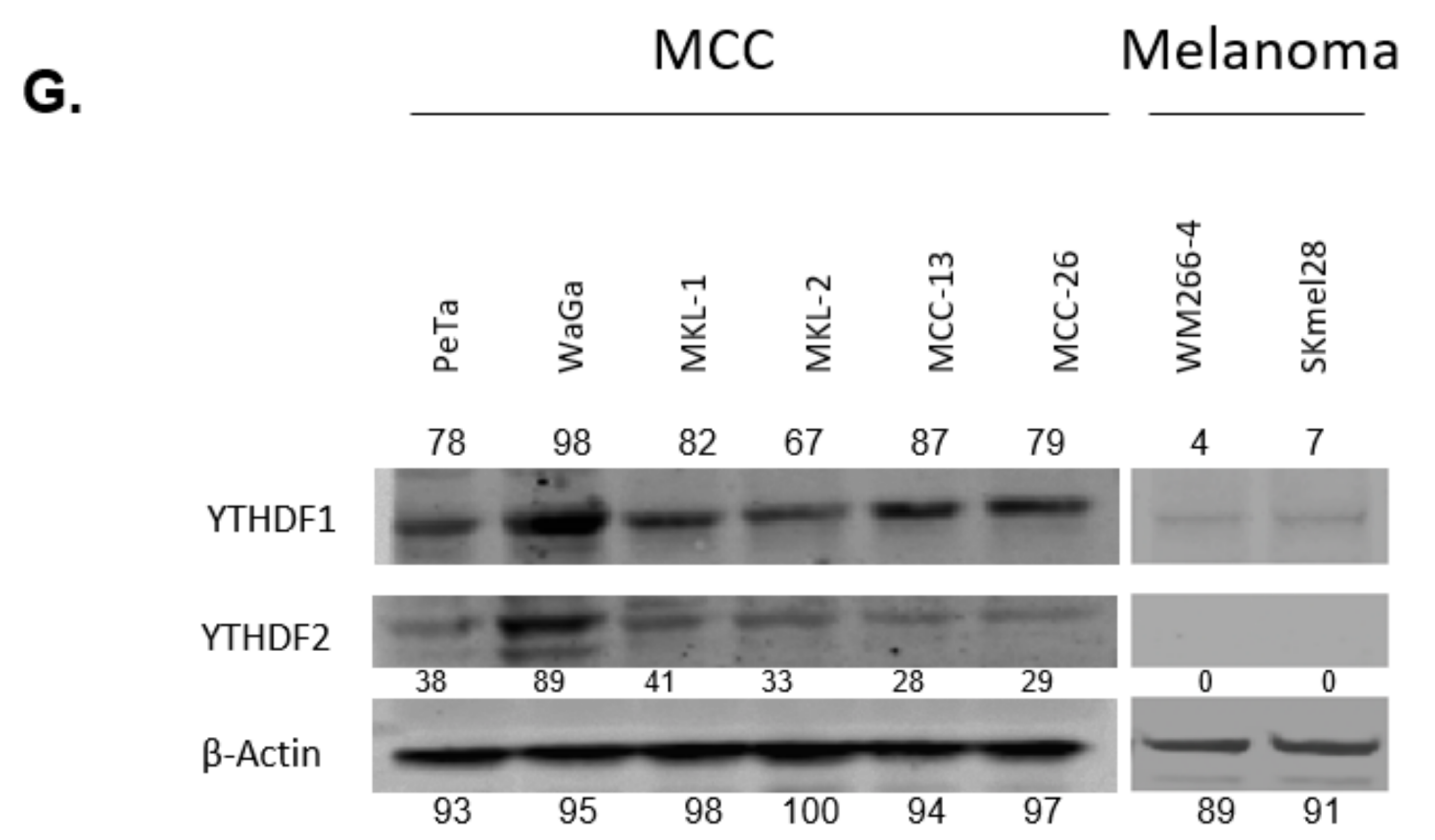
(**A**) Schematic overview of copy number variation and gene expression analysis, comparison of gene expression data with other types of skin cancer in the Cancer Cell Line Encyclopedia (CCLE), and validation process of the findings. (**B**) Intersection of genes with high copy numbers and highly expressing genes across all tested Merkel cell carcinoma (MCC) cell lines (FDR < 0.05). (**C**) Copy number gain in YTHDF1 and adjacent loci across all MCC cell lines. Inset shows YTHDF1 region in PeTa cell line. (**D**) YTHDF1 and YTHDF2 gene expression in skin cancers (other than MCC) from CCLE database compared to their expression in MCC cell lines. (**E**) Fluorescent in situ hybridization (FISH) ratio in MCC cell lines (fibroblasts are used as control cells, WM226-4 and SKmel28 are melanoma cell lines). (**F**) Correlation between YTHDF1 expression and amplification in MCC cell lines. (**G**) Western blot showing the expression of YTHDF1 in several MCCs and melanoma cell lines (upper panel) and the expression of YTHDF2 in the same cell lines (middle panel). Lower panel shows the expression of the internal control, β-actin. ** *p* < 0.01.

In an unbiased approach to explore copy number changes in Merkel cell carcinoma, we performed a genome-wide screen in seven established MCC cell lines using a panel of ~300 k SNPs. Using this platform enables us to analyze structural and copy number variations throughout the whole genome. Figure 1A shows schematically the analysis workflow across MCC samples. First, we identified regions that gained gene copy numbers in each of the tested cell lines. Identified regions were overlapped in tested cell lines and shared regions across all samples were obtained (Figure S1A). Four loci on chromosomes chr7, chr16, and chr20 harboring 16 genes were identified to have copy number gain across the tested cell lines (FDR < 0.05, Figure S1A). Next, we integrated transcription data from these cell lines to the copy number data. Transcriptomic analysis was performed using the Illumina RNA microarray platform. Integration of copy number and expression datasets resulted in the identification of two genes on chromosome 20, YTHDF1 and KCNQ2 (Figure 1B and Figure S1B). Based on the genome-wide profiling, YTHDF1 and KCNQ2 genes were amplified and this was correlated to their expression levels across the tested cell lines.

Since high expression of YTHDF1 was more consistent among all tested MCC cell lines than high expression of KCNQ2, we pursued to study YTHDF1 (Figure S1B). The upper panel of Figure 1C represents YTHDF1 locus across all tested MCC cell lines and the lower inset shows YTHDF1 gene copy gain at higher resolution in one of the cell lines (PeTa). In the YTHDF family of genes both YTHDF1 and YTHDF2 (but not YTHDF3) were highly expressed in all studied MCC cell lines (Figure S1C). Therefore, we assessed expression level of YTHDF1 and its major paralog YTHDF2 across other types of skin cancer to investigate whether this family of genes is essential for all cancer cells or it is a unique feature in MCC. Expression data for these genes were retrieved for numerous skin cancer cell lines (*n* = 56) from Cancer Cell Line Encyclopedia (CCLE) database. Since Merkel cell carcinoma was not part of the CCLE project, we then normalized expression values from the CCLE dataset along with our in-house MCC expression dataset. Interestingly, a > 10-fold change in the levels of these genes was noticed in MCC cell lines compared to other skin cancer cell lines (Figure 1D). To evaluate gene copy numbers, we designed fluorescent probes to detect YTHDF1 and performed fluorescent in situ hybridization in six MCC cell lines, two melanoma cell lines, and normal diploid control cells (fibroblasts). For this purpose, we used a centromere probe located on the same chromosome as YTHDF1 (CEP20) as a control to normalize YTHDF1 signals and calculated the fluorescent in situ hybridization (FISH) ratio as described previously [27]. A FISH ratio ranging from 1.78 to 3.11 was calculated for MCC cell lines compared to 1.08 and 0.97 in melanoma cells and 0.99 in fibroblasts (Figure 1E). We next sought to investigate the correlation between gene expression and amplification (copy number gain) in MCC cell lines as plotted in Figure 1F (R^2^ = 0.87).

To see if this unique finding in MCC cells at the mRNA level holds true at the protein level, we performed immunoblots with lysates derived from MCC cell lines as well as melanoma cell lines to evaluate levels of YTHDF proteins. All tested MCC cell lines indicated higher expression level of YTHDF1 and YTHDF2 compared to the tested melanoma cell lines (Figure 1G and Figure S4).

### 3.2. YTHDF1 Is Highly Amplified and Expressed in Merkel Cell Carcinoma Tumor Biopsies

**Figure 2:**
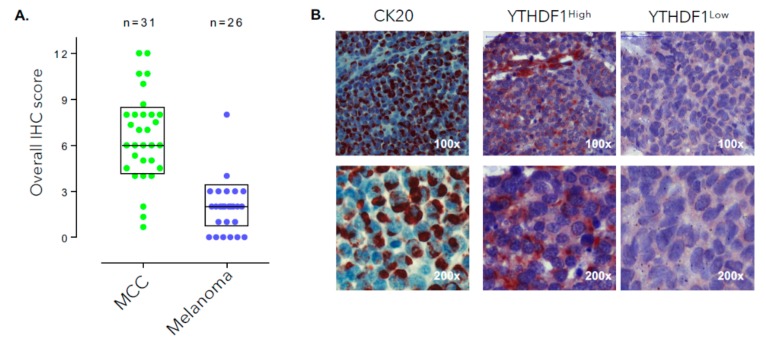

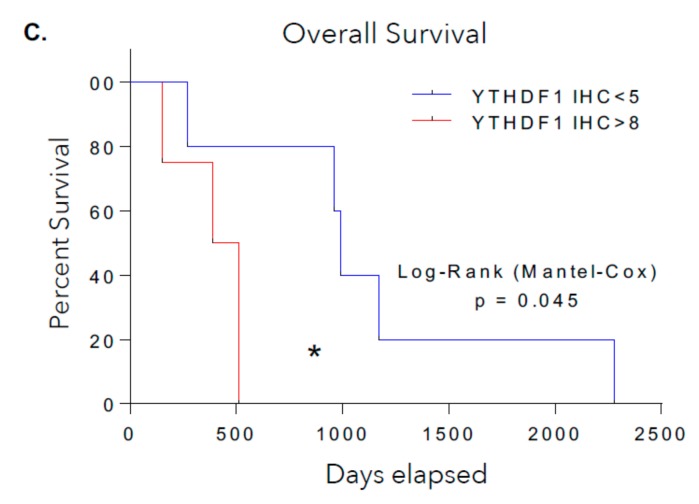
(**A**) YTHDF1 protein expression data from a cohort of 31 MCC patients compared to its expression in 26 melanoma patients. Data analyzed from tissue microarrays consisting of 3–6 replicates for each tumor sample from various sites of each tumor (total of 213 tumor cores). (**B**) Immunostaining showing expression of YTHDF1 in two samples expressing this protein at high and low level along with CK20 in a patient’s tumor specimen at different magnifications. (**C**) Comparison of overall survival data in two groups of YTHDF1^high^ (immunohistochemistry (IHC) score > 8) and YTHDF1^low^ (IHC score < 5) patient samples from those with available survival data (log-rank *p* value = 0.045), *p* < 0.05 is shown with asterisk.

To investigate whether this finding that can distinguish MCC from other skin cancers can be recapitulated in tumor sample biopsies from MCC patients, we performed YTHDF1 immunohistochemistry on tissue microarrays (TMAs) consisting of 31 MCC patients and 26 melanoma patients. Our TMAs include 3–6 cores (replicates) from different tumor sites from each of the patients (total number of 135 cores for MCC and 78 cores for melanoma were analyzed) (Figure 2A). IHC results for YTHDF1 from 31 MCC patients revealed this protein to be expressed at high levels in 23/31 (74.2%) of samples while only 1/26 (3.8%) of melanoma patients expressed YTHDF1 at high levels. Expression levels were quantified using previously described methods [25].

Cytokeratin 20 (CK20) is present in about 95% of MCC patients. Figure 2B demonstrates a representative CK20 IHC staining in MCC. Representative YTHDF1^high^ and YTHDF1^low^ MCC are shown in the right and middle panels of Figure 2B. Analysis of survival data available in our cohort of MCC patients revealed worse overall survival in YTHDF1^high^ MCC patients compared to YTHDF1^low^ patients (log-rank *p* value = 0.045) (Figure 2C and Figure S1D).

### 3.3. m^6^A RNA Modification Is Present in the Sequence of Merkel Cell Polyomavirus Transcripts in MCC Cell Lines

**Figure 3:**
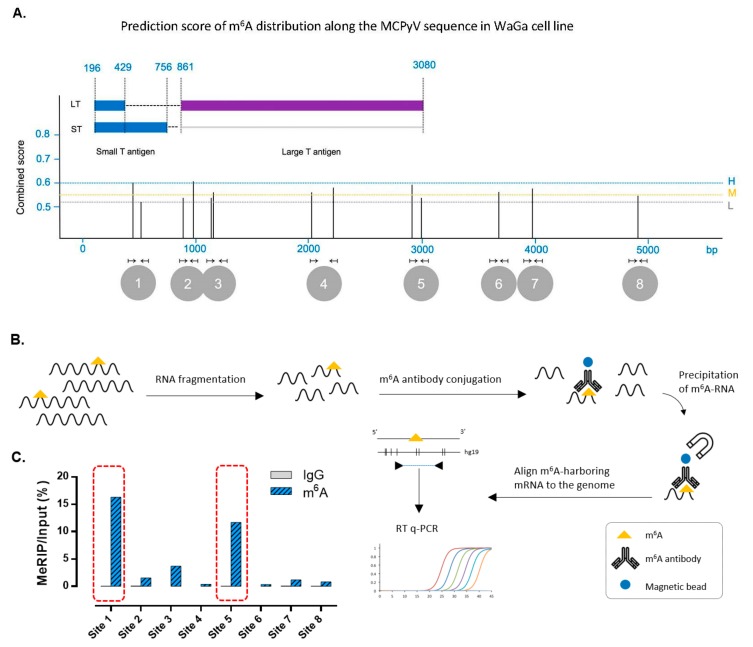
(**A**) Prediction score of m^6^A distribution along WaGa cell line according to the sequence-based RNA adenosine methylation site predictor (SRAMP) algorithm. *Y* axis shows the combined score at different levels of high (H), moderate (M), and low (L) probability. Vertical bars show the score for m^6^A sites. *X* axis shows MCPyV genome in base pair resolution. Arrows show the location of qPCR primers designed to validate m^6^A sites. Annotated features of MCPyV genome are shown above the* X* axis. (**B**) Schematic figure shows the methylated RNA immunoprecipitation (meRIP) method followed by qPCR to validate predicted m^6^A sites. (**C**) MeRIP-qPCR results show site 1 and site 5 to be amplified, indicating the presence of m^6^A in these two regions. Samples are normalized to non-crosslinked input to remove background noise. Anti-IgG antibody is used as control.

Since m^6^A modification has been reported for several viruses similar to MCPyV [28] and because YTHDF1 is a well-characterized reader of m^6^A in humans, we set out to investigate the presence of m^6^A RNA modification in mRNA derived from the integrated sequence of MCPyV in Merkel cell carcinoma. We used the sequence-based RNA adenosine methylation site predictor (SRAMP) tool to identify potential m^6^A sites in the genome of MCPyV that is integrated into the MCC cell lines [26]. SRAMP assigns a prediction score for each identified m^6^A site. Since the entire MCPyV genome (5387 bp) is integrated into the DNA of WaGa cells, we used this cell line to perform the validation experiments. SRAMP detected a total of 13 m^6^A sites in WaGa’s MCPyV sequence and the two sites with the highest probability scores were determined at positions 410 and 966 of the MCPyV sequence (Figure 3A and Figure S2). To validate these m^6^A sites, we performed the methylated RNA immunoprecipitation (meRIP) method using m^6^A antibody followed by qPCR for the 13 regions predicted by the SRAMP algorithm. Specific primers were designed for the predicted m^6^A-harboring regions. Sites that were in the span <200 bp were merged into one region resulting in a total of eight sites. MeRIP followed by qPCR was performed as illustrated schematically in Figure 3B and is described in detail in Section 2. Upon normalization of m^6^A-precipitated signals to input, only two m^6^A sites (site 1 and site 5) from the eight predicted regions showed elevated levels, indicating the presence of m^6^A in the sequence of these sites (Figure 3C). Interestingly, site 1 and site 5 are located in the MCPyV-derived mRNA in the small T and large T antigen coding sequence, respectively. As a result of the splicing pattern of T antigen mRNAs [20] site 5 is also present in the different T mRNAs (Figure 3A).

### 3.4. Silencing of YTHDF1 Impacts mRNA Translation Initiation and Reduces Proliferative and Clonogenic Capacity in MCC Cells

**Figure 4:**
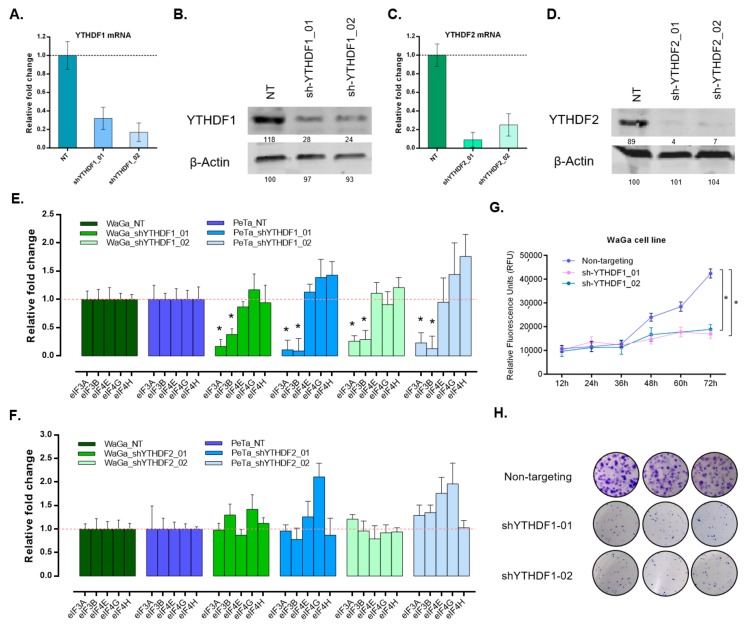
(**A**) shRNA-mediated YTHDF1 knockdown in WaGa cells. qPCR data shows the expression level of this gene in not-targeting control (NT) and two knockdown samples. (**B**) Western blot validation of YTHDF1 knockdown in WaGa cell line. β-actin is used as control. (**C**) shRNA-mediated YTHDF2 knockdown in WaGa cells. qPCR data shows the expression level of this gene in not-targeting control (NT) and two knockdown samples. (**D**) Western blot validation of YTHDF2 knockdown in WaGa cell line. β-actin is used as control. (**E**) Impact of YTHDF1 knockdown in a panel of translation initiation factors including, eIF3A, eIF3B, eIF4E, eIF4G, and eIF4H in two MCC cell lines, WaGa and PeTa. eIF3A and 3B levels are decreased significantly compared to NT controls. Asterisks indicate *p* < 0.05. (**F**) Impact of YTHDF2 knockdown in a panel of translation initiation factors including, eIF3A, eIF3B, eIF4E, eIF4G, and eIF4H in two MCC cell lines, WaGa and PeTa. No significant changes are observed. (**G**) Decreased cell proliferation rates are observed in both YTHDF1 knockdowns of WaGa compared to NT control. Asterisks indicate *p* < 0.05. (**H**) Clonogenicity capacity is reduced upon YTHDF1 knockdown in WaGa cells. Whole western blots see Figure S4.

To elucidate the role of YTHDF proteins, their encoding genes were silenced using shRNA-mediated knockdown. Two MCC cell lines (WaGa and PeTa) were silenced for YTHDF1 as well as YTHDF2 (Figure 4A–D and Figure S3A) using two shRNAs. Due to the previously known role of m^6^A modification in translation efficiency [16], we assessed if translational initiation factors (TIFs) are affected upon YTHDF depletion, and examined expression level in a panel of five major TIFs. Interestingly, eIF3A and eIF3B were significantly downregulated upon YTHDF1 depletion (Figure 4E). However, examined TIFs did not show any significant change in expression in YTHDF2-silenced cells compared to the control (Figure 4F).

To further investigate the impact of YTHDF1 on MCC cell phenotype, we performed cell proliferation assays in MCC cell lines. Our findings revealed that YTHDF1-deficient cells are less proliferative compared to control cells (Figure 4G and Figure S3B). Then, we performed colony formation assay on MCC cells that also revealed less clonogenic potential of YTHDF1-silenced cells compared to the control (Figure 4H). Gene set enrichment analysis (GSEA) in the MCC cell lines also revealed the Gene Ontology (GO) term, ‘translational initiation’ pathway, to be enriched in MCPyV-positive cells compared to MCPyV-negative cells (Normalized enrichment score, NES = 1.15, *p* = 0.043) which is in line with our findings. (Figure S3C,D).

## 4. Discussion

Presence of m^6^A modification has been shown in several types of cancer including brain, breast, leukemia, and lung. The impact of m^6^A modification on tumorigenesis in various types of cancer is exposed through deregulation of different components of m^6^A machinery, such as upregulation of ALKBH5 in glioblastoma stem cells as an indication of poor prognosis, or oncogenic role of FTO in leukemic cell transformation [29,30]. However, to date, no such regulatory mechanism that involves m^6^A modification is established in Merkel cell carcinoma. In this study, we demonstrate upregulation of YTHDF1 (m^6^A reader) by a gene copy gain mechanism, and the presence of m^6^A in multiple sites of MCPyV sequence in Merkel cell carcinoma that has not been reported so far.

Initial experiments were conducted in established MCC cell lines. Interestingly, all the tested cell lines harbored YTHDF1 copy gain. Significant higher expression level of this gene in MCC versus other skin cancers was also observed in cell line models. We further validated this unique finding by experiments investigating human tumor specimens.

Previous studies have shown the role of YTHDF1 in cap-dependent translation. YTHDF1 was reported to promote mRNA translation efficiency by enhancing ribosome loading on m^6^A containing DNA, which will lead to the modulation of the translation dynamics of m^6^A-modified mRNAs [16]. While Shuda et al. demonstrated a function of the small T protein in regulating cap dependent translation our results suggest that small T itself is prone to enhance cap-dependent translation promoted by m^6^A marks and YTHDF1 proteins, which we found to be highly expressed in MCC due to gene amplification [3].

**Figure 5:**
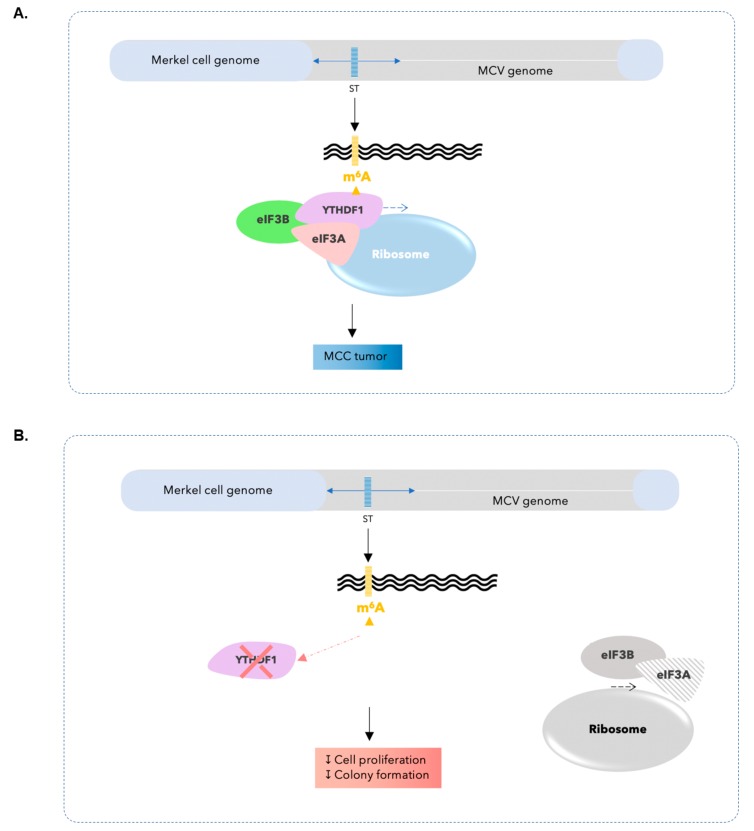
(**A**) A schematic figure demonstrating the identification of m^6^A modification in MCPyV transcriptome by YTHDF1 and further recruitment of eIF3A and 3B leading to the initiation of translation and MCC tumor. (**B**) YTHDF1 knockdown leads to lower eIF3A and 3B and lower levels of cell proliferation and clonogenicity.

Cap-dependent activation as a consequence of the presence of m^6^A marks instigates translation. Translational initiation could occur through eukaryotic initiation factors that facilitate forming the complex between mRNA and ribosomes. Here we show that in MCC cells, translational initiation regulated by YTHDF1 might happen through eIF3A and 3B, hence transforming cells to those with highly proliferative and clonogenic capabilities and turning them to neoplastic cells (Figure 5A). Silencing YTHDF1 causes a significant decrease in the expression of eIF3A and 3B in MCC cells. Further, lower expression levels of YTHDF1, m^6^A reader as well as eIF3A and 3B lead to lower proliferation and colony formation capacity in MCC cells.

Overall, our study could establish a novel tumorigenic mechanism in fatal skin cancer, Merkel cell carcinoma, that happens due to the upregulation YTHDF1 m^6^A reader, and by activation of translational initiation factors eIF3A and 3B. Interestingly, in our studied patient cohort, higher expression levels of YTHDF1 translated to worse prognosis and survival. Therefore, targeting YTHDF1 directly or through its binding to the translational initiation factors might be able to prevent activation of translation and subsequent products of integration of virus genome in the tumor cells. Editing m^6^A within the integrated virus genome using clustered regularly interspaced short palindromic repeats (CRISPR-Cas) technology could also be an alternative method to prevent recruitment of YTHDF1 leading to MCC tumorigenesis. In addition, there is definitely a need for further validation of these findings using in vivo experiments such as the use of mouse models harboring transgenic cassette with K5 promoter to target expression of sTAg to the epidermis (K5-sTAg-IRES-tdTomato) [31,32] or using YTHDF1^lox/lox^ mouse models [33] to further investigate YTHDF1 role through m^6^A identification in tumor formation and progression. Survival studies could also be expanded to larger clinicals cohorts, and m^6^A mapping can be performed at the genome-wide level. This study is the first step in elucidating the role of YTHDF1 m^6^A reader and introducing a novel mechanism of tumorigenesis in Merkel cell carcinoma through the activation of cap-dependent translation.

## 5. Conclusions

This study underscores the role of a m^6^A reader, YTHDF1 in Merkel cell carcinoma. We demonstrate that YTHDF1 is upregulated by a copy number gain mechanism and is highly expressed in MCC cell line models as well as MCC patients. We have identified and validated the presence of m^6^A at multiple sites of the MCPyV sequence in MCC. As the result of the presence of m^6^A modification, upregulated YTHDF1 activates cap-dependent translation. Translational initiation occurs in coordination with eIF3A and eIF3B and might lead to highly tumorigenic cell features. We further show a positive correlation between the expression level of YTHDF1 and worse prognosis and overall survival of the patients investigated in this study.

## Supplementary Materials

The following are available online at https://www.mdpi.com/2072-6694/12/1/202/s1, Figure S1: supplementary data corresponding to Figure 1, Figure S2: supplementary data corresponding to Figure 3, Figure S3: supplementary data corresponding to Figure 4, Figure S4. Whole blot for Figure 1 and Figure 4.
