# Supplementary material for: Oncogenic Role of an Epigenetic Reader of m6A RNA Modification: YTHDF1 in Merkel Cell Carcinoma
Source: Cancers (Basel). 2020 Jan 14;12(1):202. doi: 10.3390/cancers12010202 (PMC7016651; doi:10.3390/cancers12010202)
Supplement: Supplementary file 1 [file cancers-12-00202-s001.pdf]

Oncogenic Role of an Epigenetic Reader of m⁶A RNA Modification, YTHDF1 in Merkel Cell Carcinoma

Elias Orouji, Wiebke K. Peitsch, Azadeh Orouji, Roland Houben and Jochen Utikal

A.

Genes and miRNAs with CN gain	Gene symbol	Region	Cytoband	Q value
Genes	HBM	Chr. 16	p13.3	<0.000
	HBA2	Chr. 16	p13.3	
	HBA1	Chr. 16	p13.3	
	RBBP8NL	Chr. 20	q13.33	0.006
	YTHDF1	Chr. 20	q13.33	
	BIRC7	Chr. 20	q13.33	
	NKAIN4	Chr. 20	q13.33	
	FLJ16779	Chr. 20	q13.33	
	ARFGAP9	Chr. 20	q13.33	
	COL20A1	Chr. 20	q13.33	
	CHRNA4	Chr. 20	q13.33	
	KCNQ2	Chr. 20	q13.33	
	LOC100130587	Chr. 20	q13.33	
	No genes *	Chr. 07	q11.1	0.023
miRNAs	MIR124-3	Chr. 20	q13.33	0.006
	MIR3196	Chr. 20	q13.33	
	MIR4326	Chr. 20	q13.33	

* This region has copy number gains but it encompasses no genes.

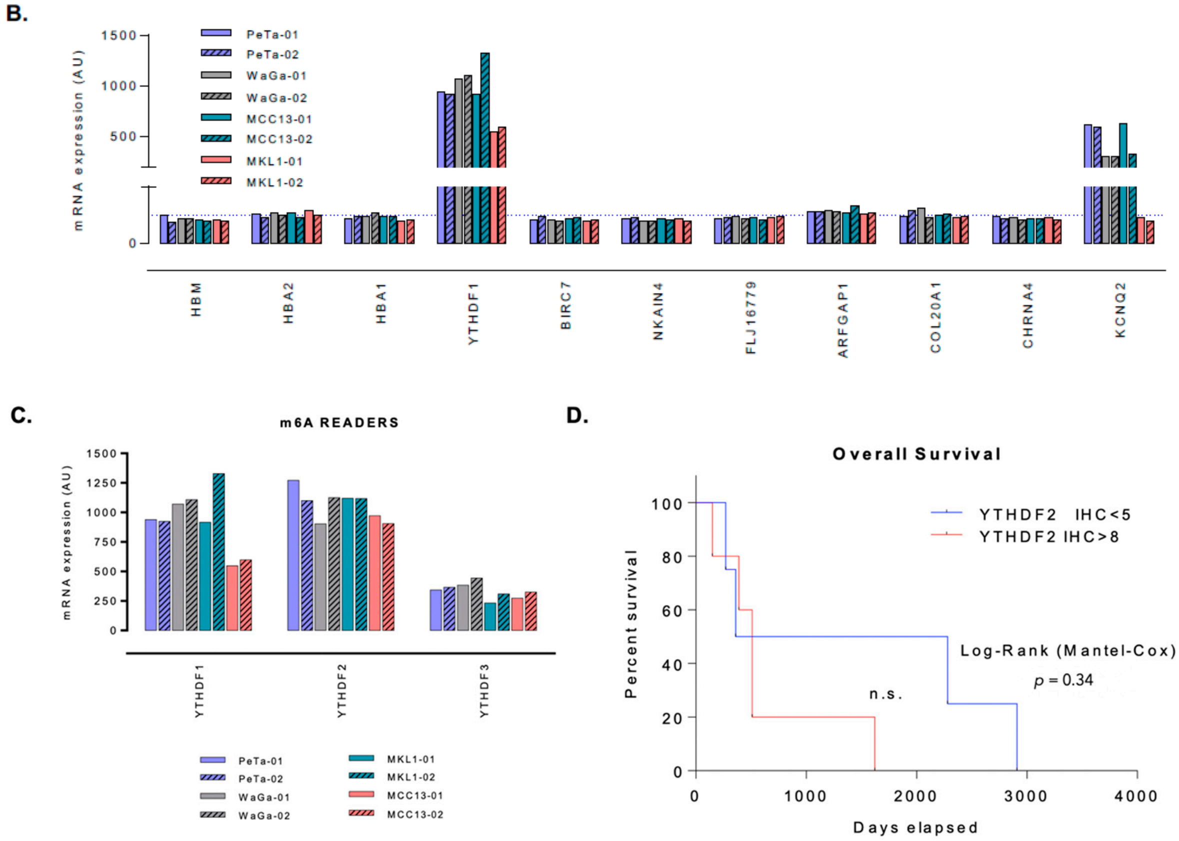

Figure S1. (**A**) Genes and miRNAs with copy number gain across all MCC cell lines (FDR < 0.05). (**B**) mRNA expression levels in genes with high copy number gains in MCC cell lines. Y axis has the arbitrary units (AU) of the fluorescence intensity. (**C**) mRNA expression in members of YTHDF gene family, YTHDF1, YTHDF2 and YTHDF3 across MCC cell lines. (**D**) Overall survival data in YTHDF2^{high} (IHC > 8) versus YTHDF2^{low} (IHC < 5) in MCC cohort (Log-Rank *p* value = 0.34).

Site #	m^6A	Position	Sequence context	Score (binary)	Score (knn)	Score (spectrum)	Score (combined)	Probability
1	1	410	AAUAAUCCACAAGCUC AGAAGUGACUUCUCU AUGUUUGAUGAGGUC	0.555	0.644	0.666	0.604	High
	2	463	AUUUCCUUGGGAAGA AUAUGGAACUUUAAA GGAUUAUAUGCAAAG	0.656	0.476	0.370	0.533	Low
2	3	885	UGACGAGGCCCCUAU AUAUGGGACCACUAA AUUCAAAGAAUGGUG	0.530	0.384	0.605	0.553	Low
	4	966	AUAUGGGCCCAAUCC ACACGGGACCAACUC AAGAUCCAGAAAGCC	0.559	0.727	0.660	0.608	High
3	5	1090	UUCUCAGCGUCCCAG GCUUCAGACUCCCAG UCCAGAGGACCCGAU	0.493	0.542	0.633	0.551	Low
	6	1106	CUUCAGACUCCCAGU CCAGAGGACCCGAUA UACCUCCCGAACACC	0.514	0.536	0.643	0.567	Moderate
4	7	2023	AUUAUCUUAGCCCAU UAUCUAGACUUUGCA AAACCAUUUCCUUGC	0.611	0.575	0.520	0.573	Moderate
	8	2211	AGAGAUGCUGGAAAU GACCAGGACAGAAAU GCUAUGUAAGAAGUU	0.645	0.652	0.478	0.579	Moderate
5	9	2920	UGCUUACAAGAAGAA AUUAAAAACUGGAAG CAAAUUUUACAGAGU	0.532	0.338	0.710	0.593	Moderate
	10	2998	GAAAAUGUAGAAGCU GGUCAGGACCCUCUG CUCAAUAUUCUUAUU	0.528	0.632	0.555	0.544	Low
6	11	3631	CACUCCAUUCUCAUC UAAAAGGACAGUAGU UAGAGUAUUACUAAA	0.685	0.635	0.391	0.565	Moderate
7	12	3982	AAUACCAGCACCAUA AUCAUGAACUCUUUU CAUGUCCCAAUAAUG	0.720	0.745	0.348	0.572	Moderate
8	13	4920	GCCACUAAUGAGAAA UUUGAAAACUGUUCA GCUGUGAACCCAAGU	0.535	0.503	0.583	0.553	Low

Figure S2. This table shows the output of SRAMP algorithm performed on MCV sequence of WaGa cell lines to predict m^6A sites. Position of each of the m^6A sites in MCV genome as well as binary, KNN (K-nearest neighbors), spectrum and combined score are shown.

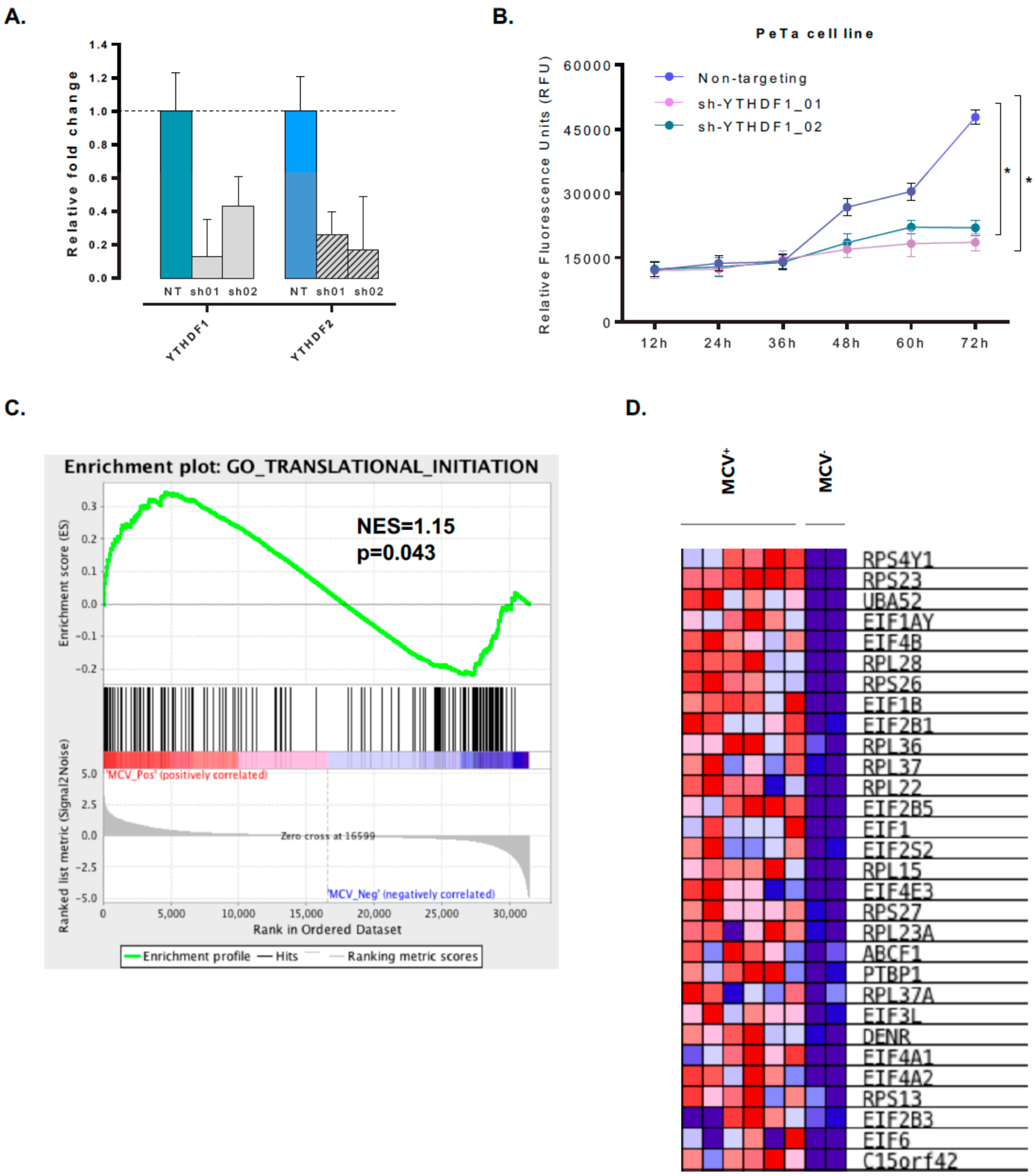

Figure S3. (**A**) qPCR indicating knock down efficiency in PeTa cell line. Non-targeting shRNA is used as control. (**B**) Cell proliferation rate is reduced upon YTHDF1 knockdown in PeTa cells. Asterisks indicates $p < 0.05$. **(C)** GSEA plot shows enrichment of GO term: Translational_Initiation in MCV^{positive} cells versus MCV^{negative} cells (NES = 1.15, p = 0.043). **(D)** Heatmap shows gene set of translation initiation factors and their expression in MCV^{positive} and MCV^{negative} MCC samples.

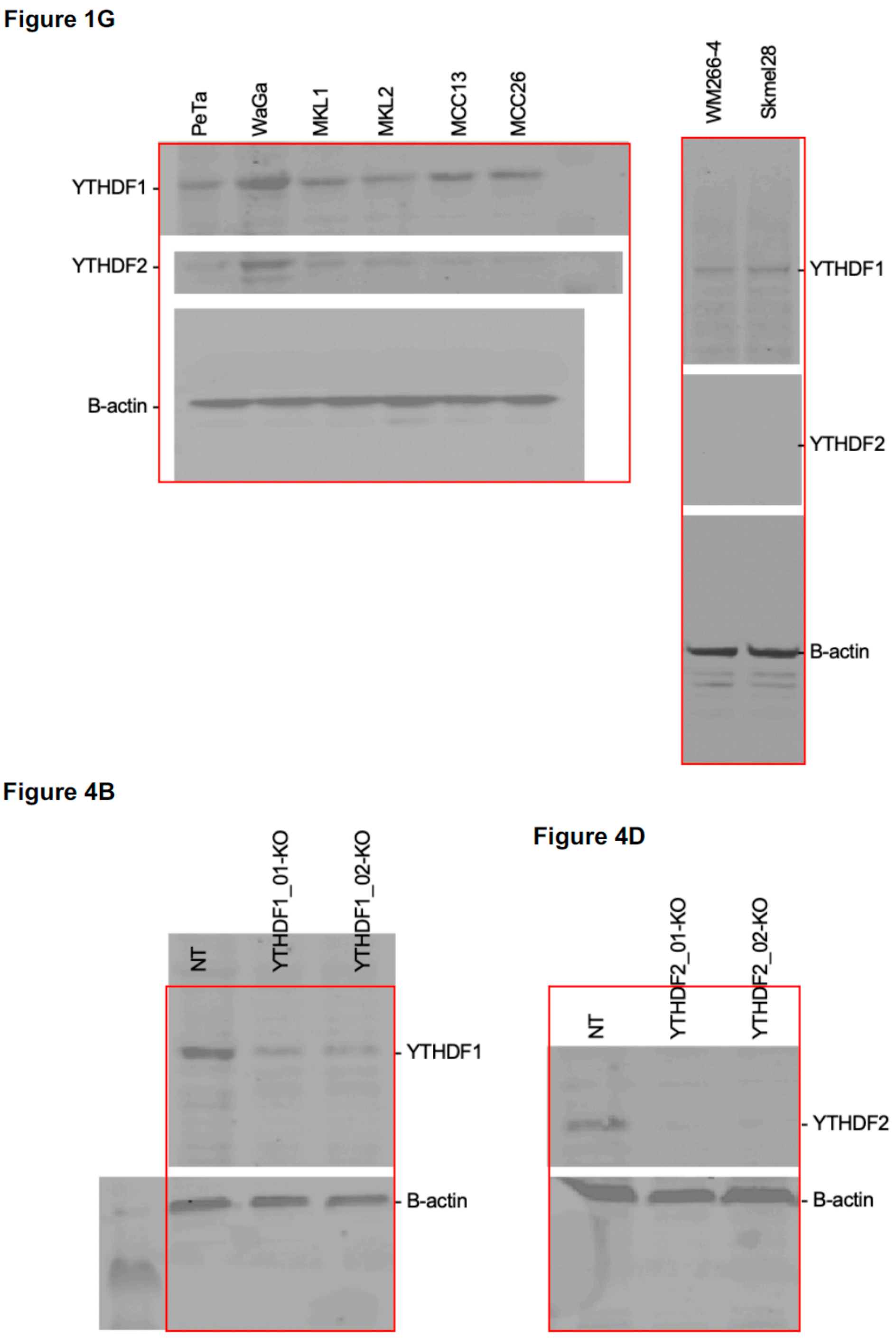

Figure S4. Whole blot for Figure 1 and Figure 4.

© 2020 by the authors. Licensee MDPI, Basel, Switzerland. This article is an open access article distributed under the terms and conditions of the Creative Commons Attribution (CC BY) license (http://creativecommons.org/licenses/by/4.0/).
